# Supplementary material for: Machine Learned Classification of Ligand Intrinsic Activity at Human μ-Opioid Receptor
Source: bioRxiv. 2024 Apr 10:2024.04.07.588485. Preprint. [Version 1] doi: 10.1101/2024.04.07.588485 (PMC11030315; doi:10.1101/2024.04.07.588485)
Supplement: Supplementary file 1 [file media-1.pdf]

Supporting Information: Machine Learned Classification of Ligand Intrinsic Activity at Human μ-Opioid Receptor

Myongin Oh,[†,‡] Maximilian Shen,[¶,§] Ruibin Liu,[‡] Lidiya Stavitskaya,[*,†] and Jana Shen[*,‡]

† *Division of Applied Regulatory Science, Office of Clinical Pharmacology, Center for Drug Evaluation and Research, United States Food and Drug Administration, Silver Spring, MD 20993, United States*

‡ *Department of Pharmaceutical Sciences, University of Maryland School of Pharmacy, Baltimore, MD 21201, United States*

¶ *Department of Electrical and Computer Engineering, University of Maryland, College Park, MD*

§*Joint first author*

E-mail: Lidiya.Stavitskaya@fda.hhs.gov; jana.shen@rx.umaryland.edu

Table S1: Summary of the evaluation metrics for the random forest and extra trees models with different feature selection thresholds

	Random Forest				Extra Trees			
α	N	AUC (%)	BA (%)	F1 (%)	N	AUC (%)	BA (%)	F1 (%)
0.2	49	91.4±3.0	83.0±4.3	74.9±6.4	49	91.1±3.6	82.3±4.7	73.6±6.7
0.3	61	91.4±3.5	82.7±4.9	74.3±7.2	61	91.1±3.1	81.5±5.0	72.9±7.4
0.4	70	91.0±3.8	83.1±5.2	74.5±7.4	69	90.4±3.5	80.7±5.0	71.3±7.5
0.5	75	91.2±3.3	81.7±5.1	72.6±7.2	76	90.3±3.6	81.7±5.1	72.8±7.3
0.6	82	91.5±3.2	82.6±5.0	73.9±6.9	82	91.3±3.4	82.7±4.7	74.7±7.1
0.7	86	91.5±3.6	82.4±5.1	73.8±7.6	86	90.5±3.6	81.3±4.9	72.0±7.2
0.8	90	91.8±2.7	82.7±4.5	74.0±6.3	90	91.5±3.9	83.3±5.0	75.4±7.2
0.9	91	91.4±3.5	82.5±4.6	74.0±6.8	91	91.2±3.3	82.6±4.8	74.3±7.1
1.0	91	91.6±3.0	82.5±3.8	74.3±5.1	91	91.0±3.4	82.5±5.0	74.1±7.4

α denotes the feature selection threshold (parameter feature_selection_threshold in PyCaret[S1]). N denotes the number of selected features. The average and standard deviations of the AUC-ROC, balanced accuracy (BA), and F1 score from the unseen tests are given.

Table S2: Description of the 10 highest ranking features in the SHAP beeswarm plot of the best performing tree-based model (ET with $\alpha = 0.8$)

**Descriptor**	**Meaning**
N_{AlHc}	Number of aliphatic heterocycles
N_{ArHc}	Number of aromatic heterocycles
N_{XCCNR}	Number of XCCNR groups
N_{ArRi}	Number of aromatic rings
N_{AlOH}	Number of aliphatic OH groups
N_{SaRi}	Number of saturated rings
N_{imd}	Number of imidazole groups
PEOE_VSA6	MOE charge VSA descriptor 6
MaxESI	Maximum electrotopological state index that combines electronic, topological, and valence states
SlogP_VSA8	MOE logP VSA descriptor 8

Table S3: Summary of the six successively optimized MPNNs built in this study

Model	1	2	3	4	5	6
Threshold optimization	×	✓	×	✓	✓	✓
Molecule-level features	×	×	✓	✓	✓	✓
Hyperparameter optimization	×	×	×	×	×	✓
k	10	10	10	10	5	5

Check mark indicates if a particular strategy, i.e., classification threshold optimization, addition of RDKit molecule-level features, or hyperparameter tuning is applied. k is the number of classifiers in an ensemble.

Table S4: Summary of the evaluation metrics for the initial and successively optimized MPNNs

**Model**	Recall(AN)	Recall(AG)	Prec(AN)	Prec(AG)	BA	F1(AN)
1	71.7±10.0	94.5±2.5	80.5±7.7	91.7±2.7	83.1±5.1	75.4±7.3
2	73.4±9.8	93.1±3.0	77.2±7.6	92.1±2.7	83.3±4.9	74.8±6.7
3	72.4±10.5	94.9±2.4	81.7±7.1	92.0±2.9	83.7±5.3	76.4±7.4
4	75.5±10.7	93.5±3.3	78.8±8.3	92.6±2.9	84.5±5.3	76.6±7.4
5	75.1±10.5	93.5±3.3	78.7±8.4	92.5±2.9	84.3±5.3	76.4±7.4
6	76.3±10.6	93.9±3.1	80.2±8.2	93.0±2.9	85.1±5.0	77.5±6.7

The average and standard deviations of the metrics from 50 data splitting and training/CV runs are given in percentage. The models are explained in Table S3.

Table S5: Summary of the predictions by the ET and D-MPNN models on the external dataset

**Compound**	**MPNN**	**ET**	**True Class**
6β-naltrexol	AN	AN	Antagonist
α-naloxol	AN	AN	Antagonist
(17S)-methylnaltrexone	AN	AN	Antagonist
Naloxazone	AN	AN	Antagonist
Apigenin	AN	AN	Antagonist
4',7-dihydroxyflavone	AN	AN	Antagonist
4'-hydroxyflavanone	~~AG~~	~~AG~~	Antagonist
PF-4455242	AN	~~AG~~	Antagonist
Bevenopran	AN	~~AG~~	Antagonist
Nalodeine	AN	~~AG~~	Antagonist
Orvinol 14	~~AG~~	AN	Antagonist
Ibogaine	AG	AG	Agonist
Dextrorphan	AG	AG	Agonist
Mitragynine	AG	AG	Agonist
Oxpheneridine	AG	AG	Agonist
Asimadoline	AG	AG	Agonist
Dermorphin	AG	AG	Agonist
3-monoacetylmorphine	AG	AG	Agonist
Eseroline	AG	AG	Agonist
$(+/-)$-salsolinol	AG	AG	Agonist
Codeinone	AG	AG	Agonist
N-methylmorphine ion	AG	AG	Agonist
Phenazocine	AG	AG	Agonist
Morphinone	AG	AG	Agonist
Zilpaterol	AG	AG	Agonist
Xylazine	AG	AG	Agonist
Morphiceptin	~~AN~~	~~AN~~	Agonist
Sophocarpidine	~~AN~~	~~AN~~	Agonist
Matrine	~~AN~~	~~AN~~	Agonist
Buprenorphine hemiadipate	AG	~~AN~~	Agonist

AG and AN stand for agonist and antagonists, respectively. Incorrect predictions are indicated with cancel signs. The true labels (19 agonists in blue and 11 antagonists in red) are taken from the work of Disney et al.,[S2] Pasternak and Pan,[S3] and NCATS Inxight drugs.[S4]

Supplemental figures

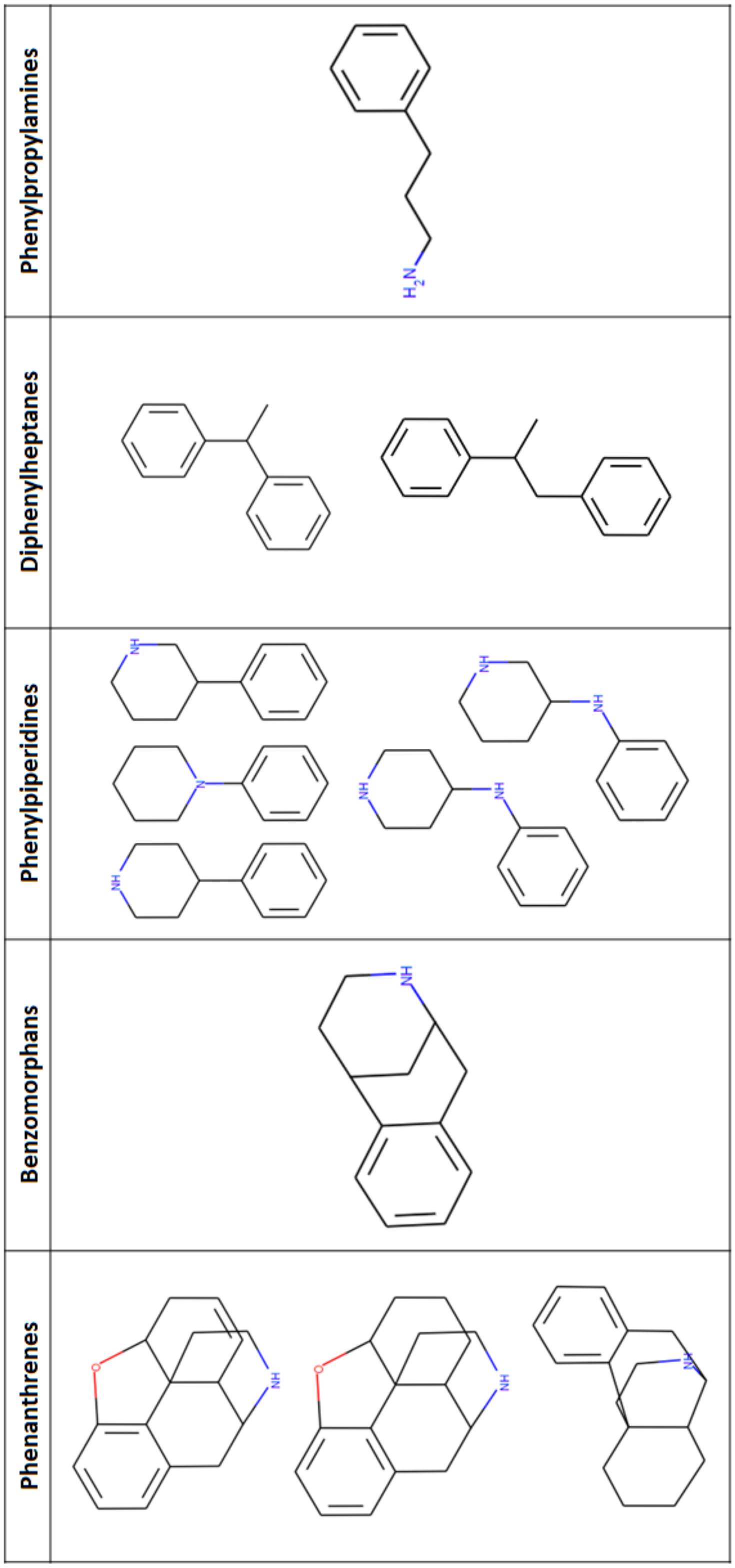

Figure S1: Common structural motifs that were manually selected for structural classification of the compounds in the training dataset

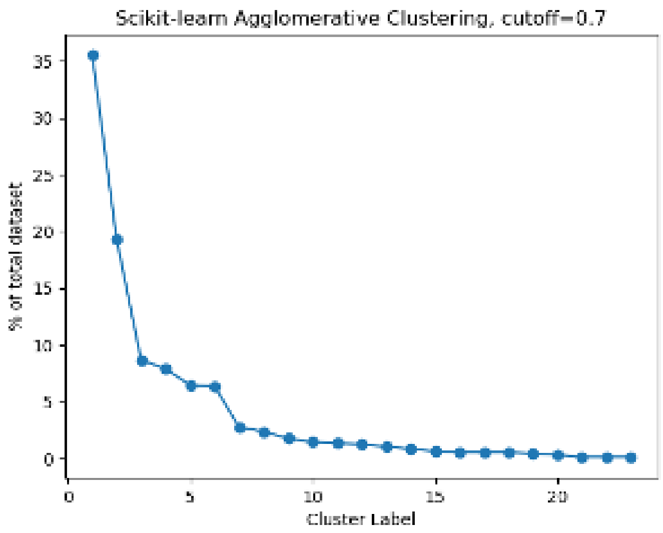

Figure S2: Cluster size vs. clustering label for the training dataset. The clustering analysis was performed on the SMILE strings of the training dataset molecules using the agglomerative method in Scikit-Learn. The clustering metric was the Tanimoto distance (1-Tanimoto similarity) of 0.7 between two molecules.

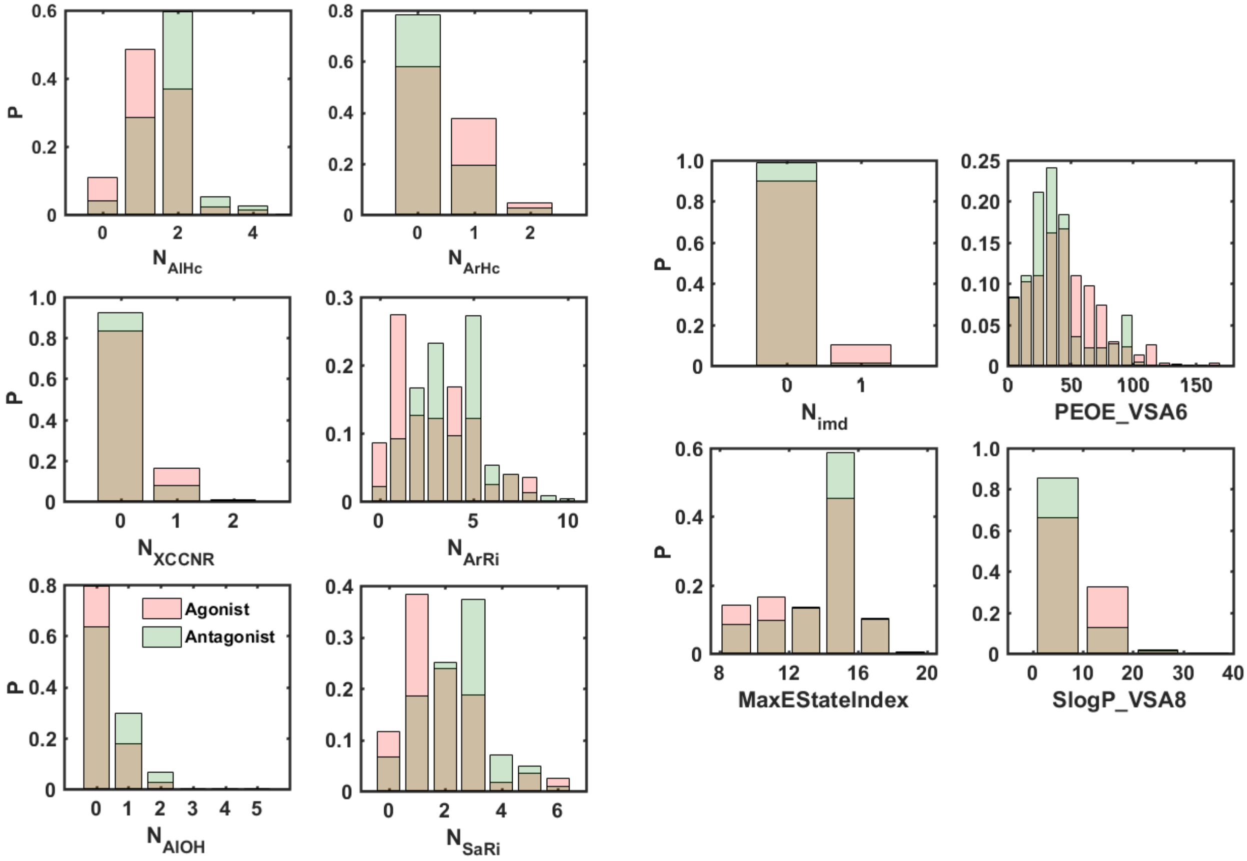

Figure S3: Normalized probability distributions of the 10 highest ranking features in the SHAP beeswarm plot of the best performing ET model for agonists (red) and antagonists (green)

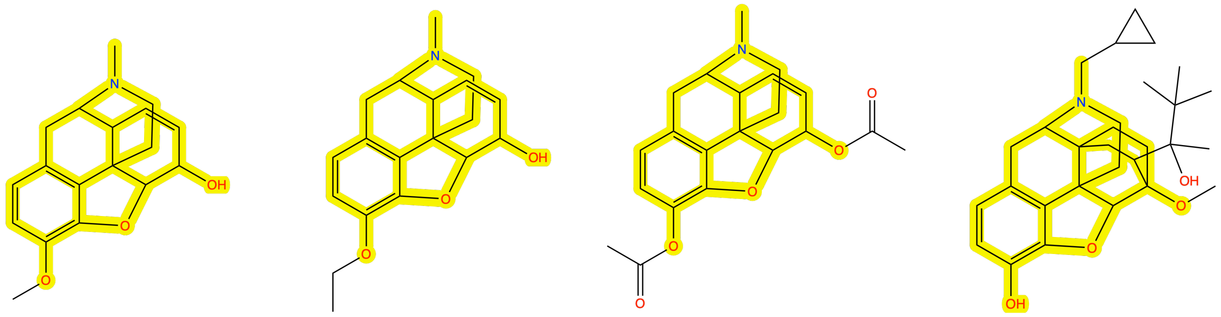

Methylmorphine (codeine) Ethylmorphine Diacetylmorphine (heroin) Buprenorphine

Figure S4: Molecular structures of some morphine derivatives in the training set. The morphine substructure is highlighted.

References

(S1) Ali, M. PyCaret: An Open Source, Low-Code Machine Learning Library in Python. 2020.

(S2) Disney, A.; Olson, K. M.; Shafer, A. M.; Moore, S. C.; Anand, J. P.; Traynor, J. R.; Husbands, S. M. Opioid Antagonists from the Orvinol Series as Potential Reversal Agents for Opioid Overdose. *ACS Chem. Neurosci.* **2022**, *13*, 3108–3117.

(S3) Pasternak, G. W.; Pan, Y.-X. Mu Opioids and Their Receptors: Evolution of a Concept. *Pharmacol. Rev.* **2013**, *65*, 1257–1317.

(S4) Siramshetty, V. B.; Grishagin, I.; Nguyen, D.-T.; Peryea, T.; Skovpen, Y.; Stroganov, O.; Katzel, D.; Sheils, T.; Jadhav, A.; Mathé, E. A.; Southall, N. T. NCATS Inxight Drugs: A Comprehensive and Curated Portal for Translational Research. *Nucleic Acids Res.* **2022**, *50*, D1307–D1316.
